# Evaluation of risk factors and correlation in large sample from the perspective of hypoglycemia

**DOI:** 10.1002/fsn3.2608

**Published:** 2021-10-13

**Authors:** Guanqun Chao, Yue Zhu, Liying Chen

**Affiliations:** ^1^ Department of General Practice Sir Run Run Shaw Hospital Zhejiang University Hangzhou China

**Keywords:** analysis, blood pressure, hypoglycemia, risk factor, triglyceride

## Abstract

To further clarify the correlation between glucose and other biochemical indicators, to search the risk factors of hypoglycemia through a cross‐sectional study. Data were obtained from subjects who underwent health examination in the Health Promotion Center from July 2018 to June 2019. Hypoglycemia is defined as fasting blood glucose less than 4.2 mmol/L. All data were analyzed using Windows R software. A total of 23,935 subjects were included. There were significant differences in age, BMI, waist circumference, blood pressure, glucose, lipid, thyroid function, liver function, and kidney function between men and women (*p *< .05). The occurrence of hypoglycemia was affected by gender and nondiabetic patients (*p* < .001). In nondiabetic patients, hypoglycemia was affected by age (*p* < .05). For each year of age increase, the risk of hypoglycemia was reduced by 2.6%; the risk of hypoglycemia decreased by 3.7% when the waist circumference increased by 1 cm; the risk of hypoglycemia increased by 30.3% with each unit of TG increased; the risk of hypoglycemia decreased by 66.1% for each unit increased by TC; the risk of hypoglycemia increased by 1.715 times for each increased unit of HDLC; the risk of hypoglycemia increased by 1.185 times for each unit of LDLC. Women and nondiabetic people are more likely to have hypoglycemia. Among the group without diabetes, diastolic blood pressure, triglyceride, HDLC, and LDLC are risk factors of hypoglycemia.

## Graphical abstract

To further clarify the correlation between glucose and other biochemical indicators, to search the risk factors of hypoglycemia through cross‐sectional study. Women and nondiabetic people are more likely to have hypoglycemia. Among the group without diabetes, diastolic blood pressure, triglyceride, HDLC, and LDLC are risk factors of hypoglycemia.

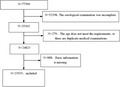

## 1 INTRODUCTION

Hypoglycemia is defined several types, including severe hypoglycemia, symptomatic, and asymptomatic hypoglycemia (all blood glucose ≤70 mg/dl, but the symptoms are different), which may be hypoglycemia (symptomatic, but blood glucose is unknown) and relative hypoglycemia (symptomatic but still more than 70 mg/dl) (Smith et al., 2018). The incidence of hypoglycemia has not been significantly lower in diabetic patients with improved blood glucose management and increased use of insulin (Holt, 2019). Studies suggest that hypoglycemia is directly associated with the risk of cognitive impairment and physical weakness (Abdelhafiz & Sinclair, 2019). Stable blood glucose concentration can ensure that the right amount of carbohydrates are transported to the brain, which may be the most fundamental reason for affecting cognition. Hypoglycemia is considered to be an interdisciplinary problem, which can affect the medication of diabetes and is related to a variety of endocrine system diseases and tumors (Rokicka et al., 2019).

With the improvement of living standards, people pay more and more attention to the physical examinations every year. In the same way, blood glucose is a must. In the physical examination results, in addition to the results of increased blood glucose, there are many physical examinations found that blood glucose is low. In the interpretation of physical examination results, physical examinees and even medical staff pay more attention to the problem of elevated blood glucose, while ignoring the decrease of blood glucose, which may lead to missed diagnosis such as islet cell tumor. Studies suggest that chronic cerebral hypoperfusion in diabetic patients is associated with recurrent hypoglycemia, cognitive impairment, and increased risk of dementia, while hypoglycemia‐related cognitive impairment is associated with cerebrovascular changes (Rehni & Dave, 2018). Chronic diseases most commonly associated with an increased risk of hypoglycemia include kidney disease, cardiovascular disease, cognitive impairment, depression, and heart failure (Silbert et al., 2018). Hypoglycemia is also increasingly recognized as a complication of weight loss therapy (Suhl et al., 2017). At present, studies on hypoglycemia are mostly focused on diabetic patients, and a large sample of healthy people has been analyzed. This study intends to analyze the relevant test results of physical examination population through the cross‐sectional study to further clarify the correlation between blood glucose and other biochemical indicators, incidence of hypoglycemia, and risk factors of hypoglycemia, so as to provide a basis for future research on blood glucose management and hypoglycemia mechanism.

## 2 MATERIALS AND METHODS

### 2.1 Data and methods

Data were obtained from subjects who underwent health examination in the Health Promotion Center from July 2018 to June 2019.

Inclusion criteria: Subjects who underwent a medical history questionnaire and blood test.

Exclusion criteria: (1) age <18 y or age >80 y; (2) pregnancy status; (3) patients with malignant tumors; (4) patients with heart disease; (5) patients with other autoimmune diseases; and (6) patients with chronic kidney or liver diseases.

### 2.2 Clinical and laboratory assessments

Clinical information was completed by trained general practitioners. Waist circumference, height,and body weight were measured by a well‐trained nurse. Body mass index (BMI) was calculated as body weight (kg)/height squared (m^2^). Systolic blood pressure and diastolic blood pressure were measured after 15 min of rest. Serum fasting blood glucose (FBG), high‐density lipoprotein cholesterol (HDL‐C), low‐density lipoprotein cholesterol (LDL‐C), total cholesterol (TC), triglyceride(TG), uric acid(UA), aminotransferase(ALT), aspartate aminotransferase (AST), blood urea nitrogen(BUN), and creatinine(Cr) were measured using a Hitachi 7600 clinical analyzer (Hitachi, Tokyo, Japan). Free triiodothyronine (FT3), free thyroxine (FT4), thyroid‐stimulating hormone (TSH), thyroid peroxidase antibody (TPOAb), and thyroid globulin antibody (TgAb) were quantified using chemiluminescent enzyme immunoassays (ICMA; Abbott, Chicago, IL, USA).

### 2.3 Diagnostic criteria

The normal range of blood glucose in our hospital was 4.2–5.8 mmol/L. Since all data are from the physical examination population, the number of people with blood glucose below 3.9 mmol/L is limited. In this study, we defined fasting glucose below 4.2 mmol/L as hypoglycemia.

### 2.4 Statistical analysis

All data were analyzed using Windows R software (version 3.5.1). Chi‐square test was used to compare categorical variables, *t*‐test or Mann–Whitney *U* test was used for continuous variable comparison. Logistic regression analysis was used to analyze the risk factors or independent predictors associated with hypoglycemia. The mean of continuous data is expressed as mean ± *SD*. *p* < .05 was statistically significant.

## 3 RESULTS

### 3.1 General information and analysis

**FIGURE 1:**
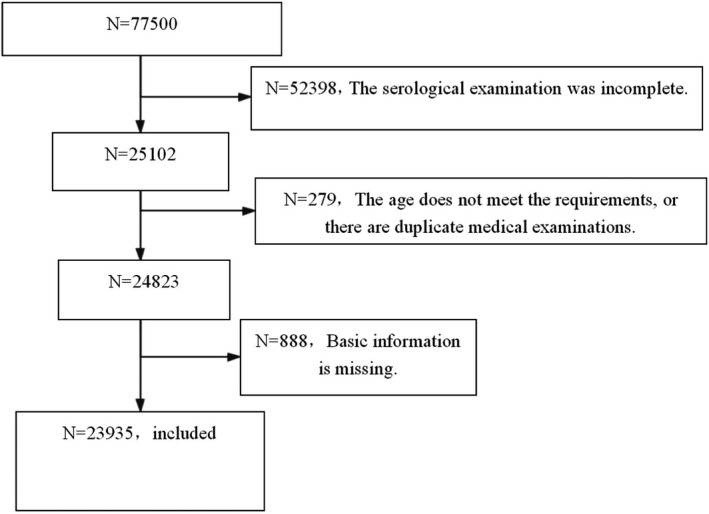
Flow diagram

According to the inclusion criteria and exclusion criteria, 23,935 subjects were included (Figure 1). Through the analysis, we found that there was significant differences in age, BMI, waist circumference, blood pressure, blood glucose, blood lipid, thyroid function, liver and kidney function between different genders. There were 13,567 males and 10,368 females. There were significant differences in age, BMI, waist circumference, blood pressure, blood glucose(fast), blood lipid, thyroid function, liver, and kidney function between men and women(*p* < .05). Among them, the age, BMI, waist circumference, blood pressure, fasting blood glucose, glycosylated hemoglobin, blood lipid (except HDLC), and liver function index of male are higher than that of female; male's uric acid is higher than female's; male's thyroxine is higher than female's; the thyroid‐stimulating hormone of female is higher than that of male; the thyroid antibody of female is higher than that of male (Table 1).

**TABLE 1:** Baseline data analysis of selected samples

	Female *N* = 10,368	Male *N* = 13,567	*p*
Age (year old)	46.92 ± 10.54	47.94 ± 10.32	<.001
BMI (kg/m^2^)	22.80 (2.99)	25.03 (3.10)	<.001
WC (cm)	77.27 (8.58)	89.20 (8.52)	<.001
SBP (mmHg)	117.44 (17.59)	124.91 (15.04)	<.001
DBP (mmHg)	69.00 (10.91)	75.59 (10.70)	<.001
Glucose (mmol/L)	5.14 ± 0.86	5.50 ± 1.33	<.001
Hba1c (%)	5.38 ± 0.60	5.56 ± 0.81	<.001
TG (mg/dl)	1.31 ± 0.98	1.99 ± 1.71	<.001
TC (mg/dl)	4.77 ± 0.93	4.83 ± 0.94	<.001
HDLC (mg/dl)	1.42 ± 0.33	1.16 ± 0.28	<.001
LDLC (mg/dl)	2.62 ± 0.73	2.74 ± 0.76	<.001
ALT (IU/L)	18.99 ± 15.26	31.20 ± 26.19	<.001
AST (IU/L)	18.58 ± 8.41	22.14 ± 13.42	<.001
UA (μmol/L)	287.27 ± 59.84	400.56 ± 78.26	<.001
BUN (mmol/L)	4.56 ± 1.14	5.06 ± 1.16	<.001
Cr (μmol/L)	57.79 ± 8.61	80.65 ± 13.24	<.001
FT3 (pg/ml)	2.80 ± 0.41	2.97 ± 0.37	<.001
FT4 (ng/dl)	1.00 ± 0.12	1.02 ± 0.11	<.001
TSH (mIU/L)	2.00 ± 1.47	1.71 ± 1.44	<.001
TGAb (IU/ml)	25.62 ± 93.61	7.73 ± 45.52	<.001
TPOAb (IU/ml)	27.42 ± 110.39	10.29 ± 64.41	<.001

Abbreviations: ALT, alanine aminotransferase; AST, aspartate aminotransferase; BP, blood pressure; BUN, blood urea nitrogen; DBP, diastolic blood pressure; FBG, fast blood glucose; HDL, high‐density lipoprotein; LDL, low‐density lipoprotein; NAFLD, nonalcoholic fatty liver disease; SBP, systolic blood pressure; SUA, serum uric acid; TC, total cholesterol; TG, triglycerides; WC, waist circumference.

### 3.2 Analysis of the correlation between blood glucose and gender, age, and other indicators

The following results were obtained by linear correlation analysis: ① fasting blood glucose was correlated with age, BMI, waist circumference, blood pressure, blood lipid, liver function index, and thyroid hormone, but the correlation coefficient was not high, while blood glucose had no correlation with FT3. ② glycosylated hemoglobin was correlated with age, blood lipid, liver function index, FT4, TGAb, but the correlation coefficient was not high, while glycosylated hemoglobin had no correlation with FT3, TSH, and TPOAb (Table 2).

**TABLE 2:** Linear correlation analysis of blood glucose and related indexes

	Glucose	*p*	Hba1c	*p*
Age	0.205	<.001	0.307	<.001
BMI	0.238	<.001	0.225	<.001
Wc	0.286	<.001	0.270	<.001
ABP	0.217	<.001	0.187	<.001
DBP	0.184	<.001	0.140	<.001
TG	0.214	<.001	0.174	<.001
TC	0.119	<.001	0.112	<.001
HDLC	−0.140	<.001	−0.146	<.001
LDLC	0.042	<.001	0.065	<.001
ALT	0.136	<.001	0.101	<.001
AST	0.083	<.001	0.060	<.001
UA	0.087	<.001	0.0.072	<.001
BUN	0.108	<.001	0.146	<.001
Cr	0.272	<.001	0.031	<.001
FT3	−0.010	.117	−0.002	.792
FT4	0.061	<.001	−0.014	.028
TSH	−0.019	.003	−0.002	.666
TGAb	−0.019	.003	−0.017	.009
TPOAb	−0.015	.023	−0.007	.248

Note

*p* >.05 means no correlation.

Abbreviations: ALT, alanine aminotransferase; AST, aspartate aminotransferase; BP, blood pressure; BUN, blood urea nitrogen; DBP, diastolic blood pressure; FBG, fast blood glucose; HDL, high‐density lipoprotein; LDL, low‐density lipoprotein; NAFLD, nonalcoholic fatty liver disease; SBP, systolic blood pressure; SUA, serum uric acid; TC, total cholesterol; TG, triglycerides; WC, waist circumference.

### 3.3 Relationship between hypoglycemia (blood glucose below normal range) and gender, diabetes mellitus

We found that: (1) The incidence of hypoglycemia was affected by gender (*p* < .001), and the possibility of hypoglycemia (<4.2 mmol/L) in women was significantly higher than that in men. (2) Hypoglycemia was affected by diabetic and nondiabetic patients (*p* < .001). The possibility of hypoglycemia (<4.2 mmol/L) in diabetic patients was significantly lower than that in nondiabetic patients (Table 3).

**TABLE 3:** Relationship between hypoglycemia and gender, diabetes mellitus

	Female *N* = 10,368	Male *N* = 13,567	*p*	NonDiabetes *N* = 22,721	Diabetes *N* = 1214	*p*
Non‐hypoglycemia	10,173	13,395	<.001	22,359	1209	.002
Hypoglycemia	195	172		362	5	

Note

*p* < .05, indicating statistical significance.

### 3.4 Analysis of the correlation between the frequency of hypoglycemia and age

Since the theoretical frequency is less than 1, Fisher's exact test is used in this study. The results showed that: (1) In diabetic patients, hypoglycemia was not affected by age (*p* > .05), indicating that the possibility of hypoglycemia (<4.2 mmol/L) was similar in different age groups. (2) In nondiabetic patients, hypoglycemia was affected by age (*p* < .05), suggesting that the possibility of hypoglycemia (<4.2 mmol/L) was different in different age groups (Tables 4 and 5).

**TABLE 4:** Analysis of the correlation between the frequency of hypoglycemia and age

	~30	30	40	50	60	70~	*p*
Diabetes *N* = 1219							
Non‐hypoglycemia	7	47	238	553	314	50	.1215
Hypoglycemia	0	0	3	0	2	0	
Non‐diabetes *N* = 22,721							
Non‐hypoglycemia	1125	3950	7830	6988	2121	345	<.001
Hypoglycemia	47	97	120	84	12	2	

Note

*p* < .05, indicating statistical significance.

**TABLE 5:** Analysis of risk factors of hypoglycemia

	Age	WC	SBP	DBP	TG	TC	HDLC	LDLC
OR	0.975	0.963	0.982	1.019	1.303	0.369	2.715	2.185
*p*	<.001	<.001	.002	.025	<.001	<.001	<.001	<.001

Note

OR >1 is risk factor; OR <1 is protective factor.

### 3.5 Analysis of risk factors of hypoglycemia

There were 1219 diabetic patients. Because of the small sample size, the reference value of logistic regression was limited. There were 22,841 nondiabetic patients. Logistic regression analysis was used. Finally, the logistic model was statistically significant. Among the independent variables included in the model, age, waist circumference, systolic blood pressure, diastolic blood pressure, TG, TC, HDLC, and LDLC were statistically significant. The results showed that: (1) For each year of age increase, the risk of hypoglycemia was reduced by 2.6%; (2) the risk of hypoglycemia decreased by 3.7% when the waist circumference increased by 1 cm; (3) the risk of hypoglycemia increased by 30.3% with each unit (mg/dl) of TG increased; (4) the risk of hypoglycemia decreased by 66.1% for each unit (mg/dl) increased by TC; (5) the risk of hypoglycemia increased by 1.715 times for each unit increased unit (mg/dl) of HDLC; (6) the risk of hypoglycemia increased by 1.185 times for each unit (mg/dl) of LDLC.

## 4 DISCUSSION

Abnormal blood glucose is common in the clinic. Most hypoglycemic events are thought to be related to diabetes. However, hypoglycemia often occurs in nondiabetic patients. The occurrence of diabetes in nondiabetic inpatients is related to malnutrition, heart failure, malignant tumor, kidney disease, etc., and it is considered that the occurrence of such hypoglycemia is related to high mortality (Vihonen et al., 2018). At present, researches on the mechanism of hypoglycemia mostly focus on diabetes patients. Studies have found that alcohol can induce hypoglycemia, and the main reason is that the inhibition of hepatic gluconeogenesis downregulates the antiregulatory hormone and reduces the release of glucose from the liver, and hypothermia directly disrupts glucose homeostasis (Vihonen et al., 2018). Hypoglycemia has also been shown to be associated with the nervous system, such as epilepsy, and low blood glucose levels are considered to be an important trigger for physiological seizures (Badawy et al., 2013). Hypoglycemia caused by congestive heart failure and cardiogenic shock is associated with secondary liver failure (Kataja et al., 2017). The causes of hypoglycemia also include age, insulin, weight loss, renal function decline, and changes in diet or drugs (Mathioudakis et al., 2018). It can be seen that the occurrence of hypoglycemia is related to many factors, such as diabetes or more serious diseases, and is also related to the length of stay. However, there are few studies on the incidence of hypoglycemia in the general population.

Our study includes a large number of physical examination population, which has the characteristics of the general population and can better explain the situation of the public. Through data analysis, we found that the level of multiple indicators in men was significantly higher than that in women, including blood glucose level. In general, women's fat is distributed in the periphery, while men's fat is mostly distributed in the viscera, and women's muscle mass is often less than men's so that less muscle is used to absorb glucose (Gonzalez‐Jaramillo et al., 2019). However, our study found that the waist circumference and body mass index of men are significantly higher than those of women. It is speculated that under the current situation, men tend to increase fat significantly, while women pay more attention to weight management, which is also the current social situation, and can also be used as the reason why men's fasting blood glucose level is significantly higher than women's. In the correlation analysis, fasting blood glucose level was correlated with age, BMI, waist circumference, blood pressure, blood lipids, liver enzymes, and thyroid hormone (except FT3). In animal experiments, the response of insulin to intravenous and intestinal nonstructural carbohydrates increased with age (Rapson et al., 2018), which showed that blood glucose level was correlated with age, which was consistent with our results. One study suggested that thyroid hormone is related to lipid metabolism and insulin metabolism (Lei et al., 2019); it also confirmed the correlation between blood glucose level and thyroid hormone. Because of the correlation between blood glucose levels and various indicators, but the correlation is not high. Therefore, we further analyzed the correlation between hypoglycemia and diabetes and gender. The results showed that hypoglycemia was related to gender and diabetes, and the possibility of hypoglycemia was higher in female and nondiabetic patients. The researchers suggest that the control of blood glucose is related to gender. Women pay more attention to blood glucose control than men and have higher attendance in outpatient service (Kautzky‐Willer & Harreiter, 2017), so that women are more likely to lower their blood glucose. The common complication of diabetes is hypoglycemia, which is often related to the use of insulin or strict management of blood glucose (Ahmad et al., 2019). Therefore, the cause of hypoglycemia that is more likely to occur in nondiabetic patients may be related to the good control of blood sugar and the high level of blood sugar in diabetics. On the other hand, it shows that diabetics are not very strict in blood sugar control. Our study also found that the incidence of hypoglycemia in nondiabetic patients was affected by age, and the occurrence of hypoglycemia was higher in the age group of 40–60 years. At present, there is no research on hypoglycemia and age, and further experiments need to be designed to verify it.

Finally, we established a logistic model. Among the independent variables included in the model, age, waist circumference, systolic blood pressure, diastolic blood pressure, TG, TC, HDLC, and LDLC were statistically significant. Among the group without diabetes, age, waist circumference, systolic blood pressure, and cholesterol are protective factors of hypoglycemia, while diastolic blood pressure, triglyceride, HDLC, LDLC are risk factors of hypoglycemia. Our results show that the increase of blood lipid is related to the incidence of hypoglycemia, which may be related to the endocrine dysfunction caused by impaired glucose tolerance and dyslipidemia. At present, it is believed that the causes of hypoglycemia in patients with diabetes mellitus are related to the use of drugs, however, for patients with non‐insulin treatment, the risk factors of hypoglycemia are mostly related to diet and exercise (Silbert et al., 2018). Another study suggested that hypoglycemia health rate and blood glucose fluctuation are more likely to occur hypoglycemia (Torimoto et al., 2018). Because hypoglycemia events are difficult to capture, there is no research on hypoglycemia in normal people. Our study used the data of the physical examination population, and the definition of hypoglycemia was limited to the below normal range, which was also due to the fact that the actual symptoms could not diagnose hypoglycemia patients. Our aim is to explore the incidence and risk factors of hypoglycemia in the population. A recent study suggested that hypoglycemia is the main cause and effect factor of cardiovascular events (Hanefeld et al., 2016). Hypoglycemia is also considered to be an obstacle to glycemic control in hospitalized patients and can affect the ultimate mortality and length of stay (Ruan et al., 2019). Therefore, regardless of whether the patient is suffering from diabetes, we should pay attention to the situation of hypoglycemia do a good job in prevention and management. Our study has some limitations: (1) the definition of hypoglycemia is not rigorous; (2) the total sample size is large, but the sample size of hypoglycemia is small; (3) there is a lack of basic research basis; (4) There is no special questionnaire for hypoglycemia. Therefore, we will increase the design basic research in the later research, expand the number of hypoglycemia samples, and adopt a strict definition of hypoglycemia as far as possible.

## 5 CONCLUSION

Hypoglycemia is still present in patients with or without diabetes. In the general population, the level of fasting blood glucose in men is significantly higher than that in women. Women and nondiabetic people are more likely to have hypoglycemia. Among the group without diabetes, age, waist circumference, systolic blood pressure, and cholesterol are protective factors of hypoglycemia, while diastolic blood pressure, triglyceride, HDLC, and LDLC are risk factors of hypoglycemia.

## CONFLICTS OF INTEREST

The authors declare no potential conflicts of interest. The data can be searched by contacting the author.

## AUTHOR CONTRIBUTION

**Guanqun Chao:** Investigation (equal); Writing‐original draft (equal); Writing‐review & editing (equal). **yue zhu:** Data curation (equal); Formal analysis (equal). **liying chen:** Supervision (equal).
